# The cost of poverty for child development: The adverse impact on maltreatment, education and mental health outcomes cannot be ignored

**DOI:** 10.1177/13591045241302121

**Published:** 2024-11-20

**Authors:** Guy CM Skinner, Eilis Kennedy

**Affiliations:** 1 Department of Public Health and Primary Care, 2152 University of Cambridge, Cambridge, UK; 2 Tavistock and Portman NHS Foundation Trust, London, UK; 3 Research Department of Clinical, Educational and Health Psychology, 8964 University College London, London, UK

**Keywords:** Child maltreatment, education, child mental health, poverty, intersectionality

## Abstract

This commentary explores the ‘cost of poverty’ for child development, with particular reference to the current UK context. Specifically, it comments on the adverse impacts of poverty on child mental health, education, and maltreatment.

## Plain Language Summary

The Cost of Poverty for Child Development: Recent reports highlight the growing issue of child poverty in the UK, with over 4 million children affected, and 1 million in Northern England alone. The Joseph Rowntree Foundation reveals that 600,000 more people, half of them children, have fallen into absolute poverty. Poverty has significant impacts on children’s well-being and increasing the risk of neglect and abuse. Data shows that many children referred to authorities for neglect and abuse are under five, and in areas like Blackpool, 1 in 52 children is in care. Poverty also severely affects children’s mental health. A report from the Children’s Commissioner links poverty to higher levels of stress, anxiety, and depression in children. NHS data shows that children in households with reduced income are more likely to experience mental health issues, with 1 in 4 affected children showing probable mental disorders. Educationally, children in poverty face serious challenges. They leave school significantly behind their peers, and only 4 in 10 disadvantaged children meet basic academic standards. This disadvantage extends into poor health and job prospects. Addressing these issues requires poverty-aware practices among professionals working with children. Mental health and social care services should integrate support for financial difficulties, including poverty screenings and financial counselling. Research is needed to evaluate the effectiveness of combined financial and psychological interventions to break the cycle of poverty. By focusing on poverty-informed practices, professionals can help improve outcomes for children in education, mental health, and overall well-being, emphasising that tackling child poverty is a necessary investment for society's future.

Recent reports from numerous sources, such as the Centre for Young Lives and Child of the North, have highlighted the alarming prevalence of child poverty in the UK, with over 4 million children affected, and a staggering 1 million residing in the North of England alone (Centre for Young Lives & Child of the North, 2024). Furthermore, the Joseph Rowntree Foundation, drawing on statistics published by the UK Government, indicate a concerning trend of absolute poverty, with an additional 600,000 individuals, half of whom are children, falling below the poverty line and rising for the second year in a row (HM Government, 2024; Joseph Rowntree Foundation, 2024). The prevalence of poverty has profound impacts on children’s educational and mental health outcomes (Barnardo’s, 2022), in addition to the likelihood of maltreatment occurring (Bywaters, Skinner, & Kennedy, 2022; Skinner et al., 2022a; 2022b).

Rising Poverty is linked to the increasing rates of child maltreatment, as noted in the Children’s Charities Coalition’s “Children At The Table” report (Children At The Table, 2023). The NSPCC Helpline’s data underscores the severity of the situation, with more than ten babies and young children being referred to authorities daily due to neglect and physical abuse (Children At The Table, 2023). Shockingly, more than two in five children referred to authorities are aged five and under, with neglect and physical abuse accounting for half of these cases (Children At The Table, 2023). In Blackpool, for example, this has resulted in 1 in 52 children now being in care (Bennet et al., 2024). As poverty soars, councils are finding themselves trapped in a cycle of spending billions on looking after children at the expense of providing support for families in need (Bennet et al., 2024).

Unsurprisingly, the effects of poverty extend beyond physical well-being, deeply influencing children’s mental health. A recent report from the Children’s Commissioner states that children growing up in poverty are more likely to experience stress, anxiety, and depression (Children’s Commissioner, 2024). The NHS Digital Survey on children’s mental health services elucidates the increased risk of adverse mental health of children living in economic precarity. 1 in 5 (19.9%) 7- to 16-year-olds lived in households that experienced a reduction in household income between 2022-23. This was more than 1 in 4 (28.6%) among children with a probable mental disorder (NHS Digital, 2023). Among 17–22-year-olds with a probable mental disorder, 14.8% reported living in a household that had experienced not being able to buy enough food or using a food bank in the past year, compared with 2.1% of young people unlikely to have a mental disorder (NHS Digital, 2023).

The implications of poverty on educational outcomes of children and young people are also becoming increasingly clear (Child Poverty Action Group, 2023; Centre for Young Lives, 2024). For example, the Centre for Young Lives and Child of the North recently reported that children who experience persistent disadvantage leave school on average 22 months behind their peers and are far less likely to pass key exams in Maths and English at age 16 (Centre for Young Lives & Child of the North, 2024). Children born into families with the lowest incomes in the UK are almost 13 times more likely to experience poor health and educational outcomes by the age of 17 years Centre for Young Lives & Child of the North, 2024). Alarmingly, only 4 in 10 of the most disadvantaged pupils will reach the expected attainment at the end of their time at school (Centre for Young Lives & Child of the North, 2024).

It is therefore imperative to consider the long-term ramifications of poverty on children and young people’s maltreatment rates, mental health, and educational outcomes. The cost-benefit of financial interventions aimed at alleviating family debt and financial difficulties must be juxtaposed against the substantial long-term costs associated with educational, child maltreatment, and mental health outcomes, as well as the associated treatment, service usage, and lost employment opportunities (Conti et al., 2021; IFS, 2021; NSPCC, 2018).

Poverty and economic insecurity are global issues, transcending geographical boundaries. The UK serves as a pertinent case study, vividly illustrating the alarming surge in child maltreatment incidents and the stark decline in educational and mental health outcomes among its youth. Hence, the implications of this case study should be acknowledged and scrutinized on a broader scale.

However, to address the critical role that professionals, particularly psychologists, psychiatrists, and those working in education or social services, can play in mitigating the impact of poverty on children, it is essential to move beyond simply raising awareness (Andermann, 2016).

For professionals working with children and families, one immediate call to action is to advocate for poverty-aware practice within their services (Knifton & Inglis, 2020). For instance, advocating for and implementing mental health interventions that specifically address the trauma and stress associated with poverty (Hodgkinson et al., 2017). This could involve incorporating routine poverty screenings into assessments, ensuring that socio-economic factors are consistently considered in treatment planning, fostering open discussions about financial stressors during therapy sessions and integrating money advice workers into primary care (Andermann, 2018; Egan & Robison, 2019). Practitioners should also seek training in culturally competent and poverty-informed care, ensuring that interventions are sensitive to the unique challenges faced by low-income families (Stubbe, 2020).

For those in service development roles, prioritizing the integration of social care and mental health services could enhance early intervention strategies. This might involve designing or adapting existing models of care that combine psychological support with access to practical resources, such as food banks, financial counselling, or housing assistance (Popay et al., 2007). Service developers could also focus on expanding outreach initiatives to increase the availability of mental health services in economically disadvantaged areas (Watt, 2018), either by establishing satellite clinics, offering mobile therapy services, or utilizing digital health platforms to reach underserved communities.

Future research needs to focus on evaluating the effectiveness of financial interventions combined with psychological support in reducing child maltreatment and improving mental health and educational outcomes. Psychologists and researchers are well-positioned to conduct studies that examine how targeted interventions—such as direct financial aid, family therapy, or mental health services—can break the cycle of poverty and its associated impacts on children. There is also a growing need for longitudinal studies that examine the long-term impact of mental health interventions on children’s educational and social outcomes in the context of poverty. These efforts will provide valuable evidence to inform service planning and policy decisions, ensuring that resources are effectively allocated to maximize the well-being of children growing up in poverty. By framing their work in terms of actionable, evidence-based solutions, professionals in mental health and education can make a tangible difference, transforming the long-term prospects for children affected by poverty.

By focusing on poverty-aware practices, service integration, and targeted research, practitioners and service developers can play a crucial role in mitigating the profound effects of poverty on children’s development, and overall life chances. Addressing child poverty is not only a moral imperative but also a strategic investment in the well-being and future prospects of societies. By prioritizing policies and interventions aimed at lifting families out of poverty, we may mitigate the adverse effects on children’s educational outcomes, reduce rates of child maltreatment, and foster better mental health outcomes.
